# Contribution of Peripheral Airways Dysfunction to Poor Quality of Life in Sarcoidosis

**DOI:** 10.1016/j.chest.2025.02.036

**Published:** 2025-03-11

**Authors:** Dimitrios Toumpanakis, Konstantinos Karagiannis, Paolo Paredi, Andras Bikov, Martina Bonifazi, Harpreet K. Lota, Harpal Kalsi, Cosetta Minelli, Nikolaos Dikaios, George A. Kastis, Peter J. Barnes, Athol U. Wells, Omar S. Usmani, Elisabetta A. Renzoni

**Affiliations:** a National Heart and Lung Institute, Imperial College London and Royal Brompton Hospital, London, United Kingdom; b Interstitial Lung Disease Unit, Imperial College London and Royal Brompton Hospital, London, United Kingdom; c Manchester University NHS Foundation Trust, Manchester, United Kingdom; d Second Department of Critical Care Medicine, “Attikon” University Hospital, Medical School, National and Kapodistrian University of Athens, Athens, Greece; e Mathematics Research Centre, Academy of Athens, Athens, Greece; f Department of Biomedical Sciences and Public Health, Università Politecnica delle Marche, Ancona, Italy; g Interstitial Lung Diseases, Pleural and Bronchiectasis Unit, Marche University Hospital, Ancona, Italy

**Keywords:** exhaled nitric oxide, lung function, oscillometry, peripheral airways, quality of life, sarcoidosis

## Abstract

**Background:**

Sarcoidosis is characterized by reduced quality of life (QoL), yet QoL is correlated poorly to conventional spirometric lung function tests.

**Research Question:**

What is the relationship of a QoL measure with comprehensive lung function assessment using oscillometry in sarcoidosis?

**Study Design and Methods:**

Sixty-two patients with pulmonary sarcoidosis completed the St. George’s Respiratory Questionnaire (SGRQ), a respiratory QoL measure, and underwent lung function assessment including oscillometry, spirometry, diffusion capacity, fractional exhaled nitric oxide (Feno), and body plethysmography. Relationships of lung function parameters with SGRQ results were determined with Spearman rank coefficient (ρ), and receiver operating characteristic curves were plotted. Logistic regression and hierarchy cluster analysis of parameters from multiple lung function techniques were performed.

**Results:**

Oscillometric indices describing peripheral lung dysfunction showed significant associations with SGRQ score (resistance at 5 Hz [R5], ρ = 0.43 [*P* < .01]; R5 minus resistance at 20 Hz [R20], ρ = 0.35 [*P* < .01]; reactance at 5 Hz [X5], ρ = –0.42 [*P* < .01]; reactance area under the curve [Ax], ρ = 0.44 [*P* < .01]), whereas FVC % predicted and residual volume to total lung capacity ratio, were related weakly to SGRQ score (ρ = –0.30 [*P* = .02] and ρ = 0.30 [*P* = .02], respectively). Oscillometry reactance, measuring elastic properties of the lung, predicted an impaired QoL (area under the receiver operating characteristic curve: Ax, 0.80 [*P* < .001] and X5, 0.78 [*P* < .001]), even in patients with absence of an obstructive or restrictive spirometry pattern. Ax remained associated significantly with SGRQ score even after adjustment for FVC and Scadding stage on multivariable analysis (*P* = .005). Feno was not associated with SGRQ score. Peripheral airway function parameters (R5 minus R20, Ax, and residual volume to total lung capacity ratio) were grouped in an independent cluster, whereas X5 constituted a single cluster.

**Interpretation:**

Our results indicate that oscillometric lung function parameters, especially those of peripheral airway dysfunction, are correlated more strongly to a QoL measure than spirometry in patients with sarcoidosis.

FOR EDITORIAL COMMENT, SEE PAGE 283

**Take-Home Points**

**Study Question:** How does lung function assessment by oscillometry correlate with quality of life (QoL) in patients with sarcoidosis?

**Results:** Oscillometric indices of peripheral lung dysfunction (resistance at 5 Hz minus resistance at 20 Hz, reactance at 5 Hz [X5], and reactance area under the curve [Ax]) showed significant association with the St. George’s Respiratory Questionnaire (SGRQ) score, whereas spirometry (FVC % predicted) and body plethysmography (residual volume to total lung capacity ratio) were only weakly related to SGRQ score. Worsening oscillometry (X5) predicted impaired QoL even in patients with absence of obstructive or restrictive spirometry findings. Oscillometry (Ax) remained associated significantly with SGRQ score even after adjustment for spirometry (FVC) on multivariable analysis.

**Interpretation:** To our knowledge, our study is the first to show that oscillometry measures of peripheral lung dysfunction correlate more closely with QoL than spirometry findings in sarcoidosis, suggesting a complementary role of oscillometry in everyday lung function assessment.

Sarcoidosis is a systemic inflammatory disease of unknown cause that affects the lungs in > 90% of patients with the disease, where nonnecrotizing granulomas follow a perilymphatic distribution, with bronchocentric inflammatory, fibrotic lesions, or both involving both large and small airways.^1^ The marked heterogeneity in the pathologic pulmonary manifestations of sarcoidosis leads to variable lung function impairment patterns, including obstructive, restrictive, or mixed spirometric abnormalities.^2^^,^^3^ Patients with pulmonary sarcoidosis report breathlessness that greatly impacts their quality of life (QoL), and loss of QoL affects treatment decisions in sarcoidosis.^4^ Yet, QoL impairment often is disproportionate to conventional lung function testing impairment, such as spirometric FEV_1_ and FVC.^5^

Oscillometry is a noninvasive technique requiring minimal patient cooperation that assesses lung function during tidal breathing and enables partitioning of the mechanical properties of the lung from the large airways to the lung periphery, including the small airways.^6^^,^^7^ Oscillometry is deployed increasingly across many respiratory diseases and correlates significantly with patient-reported outcomes.^6^ Regarding other complex multisystem diseases, oscillometry outperforms spirometry in detecting pulmonary involvement in rheumatoid arthritis^8^ and correlates with QoL in patients with systemic sclerosis.^9^ Moreover, the use of an integrational approach of clustering physiologic parameters from different lung function techniques offers unique opportunities for lung function interpretation in complex and heterogenous diseases.^10^ Previously, a complementary role of oscillometry to conventional lung function testing in the physiologic assessment of sarcoidosis was suggested.^11^ However, the relationship of oscillometry with QoL in sarcoidosis is unknown.

Fraction of exhaled nitric oxide (Feno) is a noninvasive biomarker of inflammation used in several pulmonary disorders.^12^ Although inducible nitric oxide (NO) synthase expression is increased in sarcoidosis,^13^ the literature is inconclusive regarding Feno levels in sarcoidosis and their correlation with other functional outcomes,^14^, ^15^, ^16^ whereas the association of Feno to QoL in sarcoidosis is unknown.

Thus, we investigated the relationship of lung function derangement with St. George’s Respiratory Questionnaire (SGRQ) score, a respiratory QoL measure, in patients with pulmonary sarcoidosis using both oscillometry and conventional lung function testing (spirometry, body plethysmography, single-breath transfer factor). Additionally, we assessed the relationship of Feno to QoL. We hypothesized that functional physiologic and inflammatory parameters derived by oscillometry and Feno, respectively, would be associated more strongly with SGRQ score in sarcoidosis compared with conventional lung function testing.

## Study Design and Methods

### Patients

Sixty-two patients attending the Royal Brompton Hospital sarcoidosis clinics were enrolled prospectively in this cross-sectional study. To participate in the study, patients had to be adults (between 18 and 85 years of age), to be able to provide written informed consent, and to have a diagnosis of pulmonary sarcoidosis based on previously described clinical, radiologic, and histologic criteria,^17^ in compliance with the latest American Thoracic Society clinical practice guidelines.^18^ Participants provided informed consent, and the study was approved by the Hounslow and Hillingdon Research Ethics Committee (Identifier: 08/H0709/2). Exclusion criteria were any history and evidence of neuropsychiatric disease; alcohol or drug misuse or any other condition associated with poor compliance; breast feeding; or pregnancy.

### Pulmonary Function Tests

Patients completed the SGRQ and underwent pulmonary function testing (in order: oscillometry, single-breath transfer factor, and spirometry) on the same day. Body plethysmography was assessed retrospectively, with a median time between the performance of body plethysmography and the date of the study visit of 0 months (interquartile range [IQR], –6.0 to 0 months). The composite physiologic index (CPI) was calculated.^19^ Oscillometry was performed using a Jaeger Master Screen Impulse Oscillometry system, as described previously.^7^ The following parameters are reported: resistance at 5 Hz (R5), resistance at 20 Hz (R20), R5 minus R20 (R5-R20), reactance at 5 Hz, resonant frequency (Fres), and area under the reactance curve (Ax).

### Fractional Exhaled Nitric Oxide

Feno was measured using a chemiluminescence analyzer (NIOX; Aerocrine) at expiratory flow rates of 50 mL/s, 100 mL/s, 200 mL/s, and 300 mL/s, as described previously.^20^ Measurement at multiple flows allows the partition of Feno measured at 50 mL/s to central bronchial (bronchial exhaled NO [J’awNO]) and higher flows to peripheral airway or alveolar (alveolar exhaled NO) compartments.^21^ Feno measured at 50 mL/s was measured in 44 patients; the remaining participants declined. Alveolar exhaled NO and J’awNO were calculated in 37 patients; 7 patients were unable to exhale at higher flow rates.

### Quality of Life

QoL was measured with the SGRQ, which assesses symptoms, activity, and the impact of the disease, providing a total score from 0 to 100, with higher scores reflecting increased burden on QoL,^22^ and has been used previously in patients with sarcoidosis.^5^^,^^23^

### Statistical Analysis

Data are expressed as median (IQR). The Spearman rank coefficient (ρ) was used to evaluate for correlations (presented with 95% CIs), the Mann-Whitney *U* test was used for between-group comparisons, and the χ^2^ test was used for proportions, using Statistica software (StatSoft). Receiver operating characteristic (ROC) curve analysis was performed with OriginPro software (OriginLab). Multivariable logistic regression was performed with SPSS software (SPSS, Inc.) to assess the effect of spirometry, oscillometry, and Scadding stage on SGRQ score. From each lung function technique, the physiologic parameter with the strongest correlation to the SGRQ score in the univariable analysis was chosen for the multivariable regression analysis. For both the ROC curve analysis and the multivariable logistic regression, a threshold SGRQ value of 25 was used, previously proposed to indicate increased burden on QoL in patients with COPD.^24^ A *P* < .05 defined statistical significance. Hierarchy cluster analysis based on the Ward minimum variance method,^25^ and the squared Euclidean distance as the similarity measure was performed to classify lung physiologic parameters using SPSS software. Analysis was prespecified to 5 clusters, based on the common interpretation of oscillation mechanics to 3 compartments, that is, central airways, peripheral airways, and lung tissue elastance,^6^ adding a fourth factor for the effect of heterogeneities and a fifth group for nonspecific markers. The following lung function parameters were included in the analysis: spirometry (FEV_1_ % predicted), maximum expiratory flow at 25% (MEF25%), body plethysmography (total lung capacity [TLC] % predicted); residual volume (RV) to TLC ratio, single breath transfer factor (transfer factor for carbon monoxide [TLCO] % predicted), and oscillometry (R5-R20, R20, reactance at 5 Hz [X5], and Ax). Further methodology on oscillometry and cluster analysis is provided in e-Appendix 1.

## Results

### Patients

Demographics of the patients are shown in Table 1. Forty-six patients (67.7%) were receiving ≥ 1 of the following systemic immunosuppressive treatments: corticosteroids, methotrexate, azathioprine, and hydroxychloroquine.

**Table 1:** Characteristics of Patient With Sarcoidosis (N = 62)

Characteristic	Data
Age, y	53 (45-63)
Sex	
Male	32
Female	30
Ethnicity	
White	37
Afro-Caribbean	12
Asian	13
Smoking status	
Never	40
Current	1
Former	21
BMI, kg/m^2^	27.5 (23.5-31.9)
Duration from diagnosis, y	8.00 (3.00-13.00)^a^
Scadding stage	
0	8 (12.9)
1	17 (27.4)
2	15 (24.2)
3	3 (4.8)
4	19 (30.6)
Medication	
OCS	
No. (%)	42 (67.74)
Dose, mg	7.5 (5-10)
ICS	17 (27.42)
Other systemic immunosuppressive	23 (37.10)
AZA	5
MTX	5
HCQ	11
HCQ plus MTX	2
Bronchodilators	15 (24.19)
SABA	3
LABA	10
LABA and LAMA	2
Treatment regimen	
No treatment	11 (17.74)
OCS	17 (27.42)
IS	3 (4.84)
OCS and ICS	1 (1.61)
OCS, ICS, and bronchodilators	5 (8.06)
ICS and bronchodilators	5 (8.06)
OCS and IS	14 (22.58)
ICS and IS	1 (1.61)
OCS, IS, ICS, and bronchodilators	5 (8.06)
Conventional pulmonary function	
FEV_1_, % predicted	85.4 (66.8-99.0)
FVC, % predicted	98.05 (81.0-108.5)
FEV_1_ to FVC ratio	0.75 (0.67-0.80)
MEF25% predicted	38.7 (24.6-52.1)
TLCO, % predicted	68.45 (55.6-81.0)
KCO, % predicted	87.45 (75.5-98.3)
RV, % predicted	92.9 (77.0-105.0)
TLC, % predicted	92.1 (81.7-102.8)
RV to TLC ratio	34.6 (29.3-39.75)
CPI	23.2 (12.1-37.7)
Impulse oscillometry	
R5, kPa/L/s	0.43 (0.37-0.54)
R20, kPa/L/s	0.35 (0.30-0.41)
X5, kPa/L/s	–0.15 (–0.20 to –0.12])
Fres, Hz	15.83 (13.3-20.12)
AX, kPa/L	0.74 (0.35-1.26)
R5-R20, kPa/L/s	0.097 (0.05-0.13)
Exhaled nitric oxide	
Feno50, ppb^b^	21.2 (13.7-27.5)
C_A_NO, ppb^c^	3.77 (2.69-5.65)
J’awNO, pL/s^c^	925.17 (592.97-1,213.10)
Health status, quality of life	
Total SGRQ score	30.8 (13.7-44.9)
Symptoms domain	35.7 (12.8-51.3)
Activity domain	41.8 (17.9-65.9)
Impact domain	20.5 (6.9-36.6)

Data are presented as No. (%), No., or median (interquartile range). Ax = reactance area under the curve; AZA = azathioprine; C_A_NO = alveolar exhaled nitric oxide; CPI = composite physiologic index; Feno50 = fractional exhaled nitric oxide measured at 50 mL/s; Fres = resonant frequency; ICS = inhaled corticosteroid; IS = systemic immunosuppressive; J’awNO = bronchial exhaled nitric oxide; HCQ = hydroxychloroquine; KCO = carbon monoxide transfer coefficient; LABA = long acting beta2-agonist; LAMA = long acting muscarinic antagonist; MEF25% = maximum expiratory flow at 25%;OCS = oral corticosteroid; ppb = parts per billion; R5 = resistance at 5 Hz; R5-R20 = resistance at 5 Hz minus resistance at 20 Hz; R20 = resistance at 20 Hz; RV = residual volume; SABA = short acting beta2-agonist; SGRQ = St. George’s Respiratory Questionnaire; TLC = total lung capacity; TLCO = transfer factor for carbon monoxide; X5 = reactance at 5 Hz.

a Available for 45 of 62 patients.

b Forty-four of 62 patients had Feno50 measurements.

c C_A_NO and J’awNO were calculated in 37 of 62 patients.

### Spirometry, Lung Volumes, and Gas Transfer Analysis

Lung function is shown in Table 1. An obstructive pattern (defined as FEV_1_ to FVC ratio of < 0.7)^26^ was observed in 23 patients (37.1%), and a restrictive pattern (TLC less than lower limit of normal) was observed in 16 patients (26%). Five patients (8.06%) demonstrated a mixed obstructive and restrictive functional abnormality. TLCO was 68.45% predicted (IQR, 55.6%-81.0% predicted). The CPI score was 23.2 (IQR, 12.1-37.7), with 11 patients (18%) showing a CPI of > 40, associated with poor prognosis in sarcoidosis (Table 1).^17^

### Oscillometry

Oscillometry data are shown in Table 1. Thirty-nine patients (57.68%) had an R5-R20 of > 0.074, a threshold widely accepted as the upper limit of normal,^27^ denoting the existence of frequency dependence of resistance in this group and implying the presence of peripheral airway dysfunction. The reactance marker X5 (reflective of the elastic properties of the lung tissue^6^) was –0.15 kPa/L/s (IQR, –0.20 to –0.12 kPa/L/s; a value numerically more negative compared with historical data obtained from healthy participants in our department^7^). The Ax reflecting changes in the elastic properties of the respiratory system was 0.74 kPa/L (IQR, 0.35-1.26 kPa/L).

### Fractional Exhaled Nitric Oxide

Feno measured at 50 mL/s was 21.2 parts per billion (ppb; IQR, 13.7-27.5 ppb). Partitioning to central or bronchial (J’awNO) origins and peripheral (small airways or alveolar) origins (alveolar exhaled NO) revealed a level of 925.17 pL/s (IQR, 592.97-1,213.10 pL/s) and 3.77 ppb (IQR, 2.69-5.65 ppb), respectively (Table 1).

### QoL Measure

Α median total SGRQ score of 30.8 (IQR, 13.7-44.9) was found, with 34 patients (54.84%) reporting a SGRQ score of > 25. SGRQ values were not returned for 2 patients (Table 1).

### Relationship of Oscillometry With Lung Function Tests: Spirometry and Body Plethysmography

**Figure 1:**
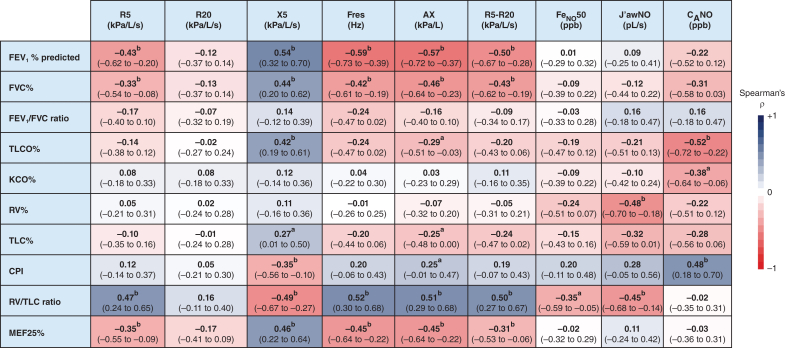
Correlation matrix between oscillometry and exhaled nitric oxide (NO) measures with conventional lung function tests parameters. A blue-to-red heatmap of Spearman rank coefficient (ρ) values is presented for each pairwise correlation, where the intensity of the color indicates the strength of the relationship (blue indicates a positive correlation and red indicates a negative correlation). Data presented as ρ (95% CI). ^a^*P* < .05. ^b^*P* < .01. Ax = reactance area under the curve; C_A_NO = alveolar exhaled nitric oxide; CPI = composite physiologic index; Feno 50 = fractional exhaled nitric oxide measured at 50 mL/s; Fres = resonant frequency; J’awNO = bronchial exhaled nitric oxide; KCO = carbon monoxide transfer coefficient; MEF25% = maximum expiratory flow at 25%; ppb = parts per billion; R5 = resistance at 5 Hz; R5-R20 = resistance at 5 Hz minus resistance at 20 Hz; R20 = resistance at 20 Hz; RV = residual volume; TLC = total lung capacity; TLCO = transfer factor for carbon monoxide; X5 = reactance at 5 Hz.

All oscillometry markers (except R20) correlated significantly with FEV_1_ and FVC and were associated moderately with increasing RV to TLC ratio, a marker of gas trapping (Fig 1). Interestingly, oscillometric parameters of X5 and Ax, indicating peripheral lung dysfunction, correlated significantly with decreasing TLCO (ρ = 0.42 [95% CI, 0.19-0.61; *P* < .01] and ρ = –0.29 [95% CI, –0.51 to –0.03; *P* = .02], respectively) (Fig 1).

### Feno Relationship With Lung Function Parameters

The alveolar component of Feno (alveolar exhaled NO) showed a significant negative relationship with TLCO (ρ = –0.52 [95% CI, –0.72 to –0.22; *P* < .01]) (Fig 1). In contrast, a weakly negative, yet statistically significant, association was noted between J’awNO and oscillometric parameters of both total and central airway resistance (R5: ρ = –0.37 [95% CI, –0.62 to –0.04; *P* = .025] and R20: ρ = –0.37 [95% CI, –0.62 to –0.04; *P* = .026], respectively) (e-Table 1). Worsening gas trapping (RV to TLC ratio) showed a negative correlation with Feno measured at 50 mL/s and its central bronchial component (J’awNO; ρ = –0.35 [95% CI, –0.59 to –0.05; *P* = .02] and ρ = –0.45 [95% CI, –0.68 to –0.14; *P* < .01], respectively).

### Relationship of Oscillometry, Feno, and Conventional Lung Function With SGRQ Score

**Figure 2:**
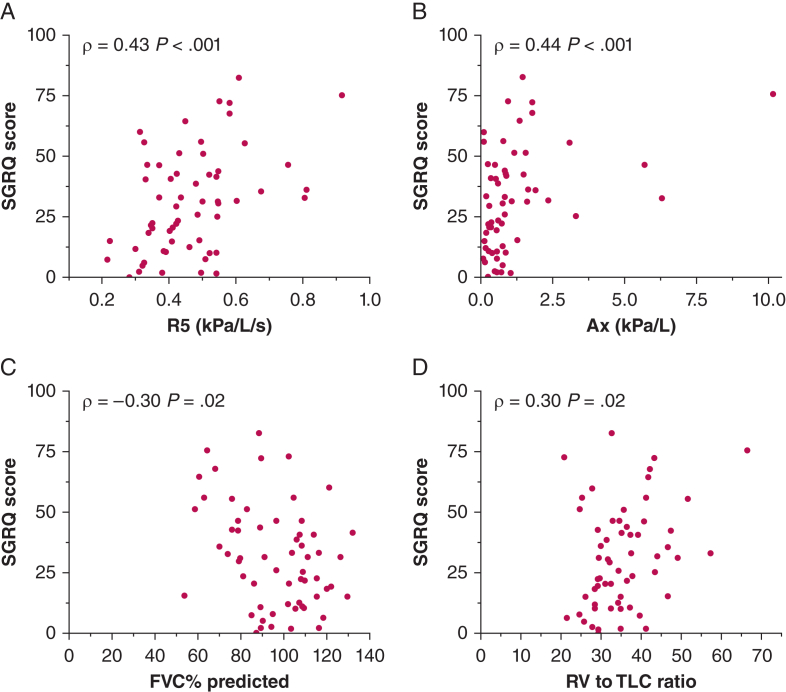
A-D, Graphs showing correlation of lung function parameters with SGRQ score in patients with sarcoidosis: R5 (A), Ax (B), FVC (C), and RV to TLC ratio (D). Evaluation of physiologic derangement with oscillometry resulted in a stronger correlation with respiratory quality of life impairment, as assessed by the total SGRQ score. R5 and Ax were the parameters correlated more strongly to SGRQ score. In contrast, conventional lung function tests showed only weak correlation, with FVC and RV to TLC ratio being the only parameters with statistically significant correlations. Ax = reactance area under the curve; R5 = resistance at 5 Hz; RV = residual volume; SGRQ = St. George’s Respiratory Questionnaire; TLC = total lung capacity.

**Figure 3:**
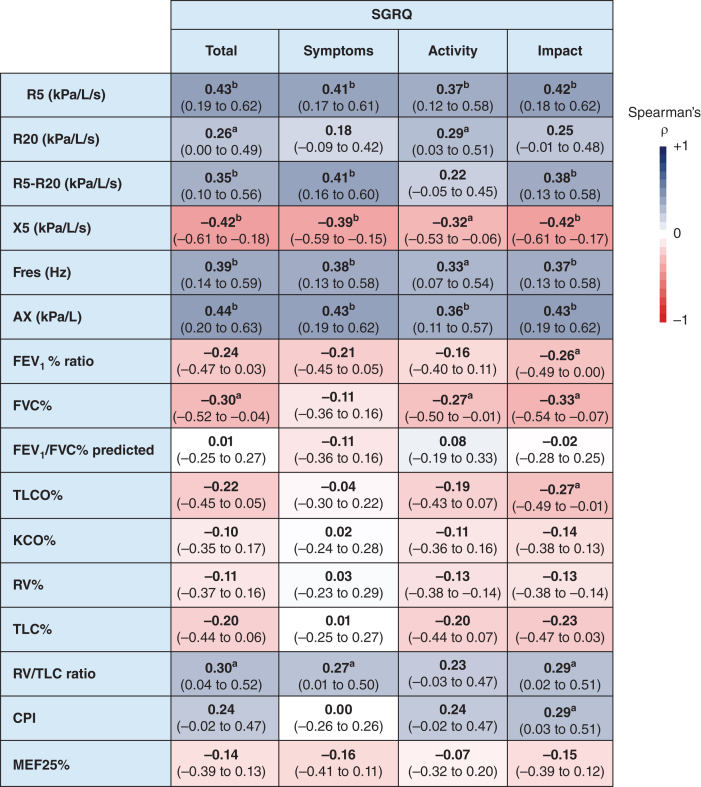
Correlation matrix between lung function parameters with respiratory quality of life, as assessed by total SGRQ score and each individual domain (symptoms, activity, impact). Strength of each pairwise correlation is visualized by a blue-to-red color scale (blue indicates positive correlation and red indicates negative correlation). Data presented as Spearman's rank coefficient (ρ) (95% CI). ^a^*P* < .05. ^b^*P* < .01. Ax = reactance area under the curve; CPI = composite physiologic index; Fres = resonant frequency; KCO = carbon monoxide transfer coefficient; MEF25% = maximum expiratory flow at 25%; R5 = resistance at 5 Hz; R5-R20 = resistance at 5 Hz minus resistance at 20 Hz; R20 = resistance at 20 Hz; RV = residual volume; SGRQ = St. George’s Respiratory Questionnaire; TLC = total lung capacity; TLCO = transfer factor for carbon monoxide; X5 = reactance at 5 Hz.

**Figure 4:**
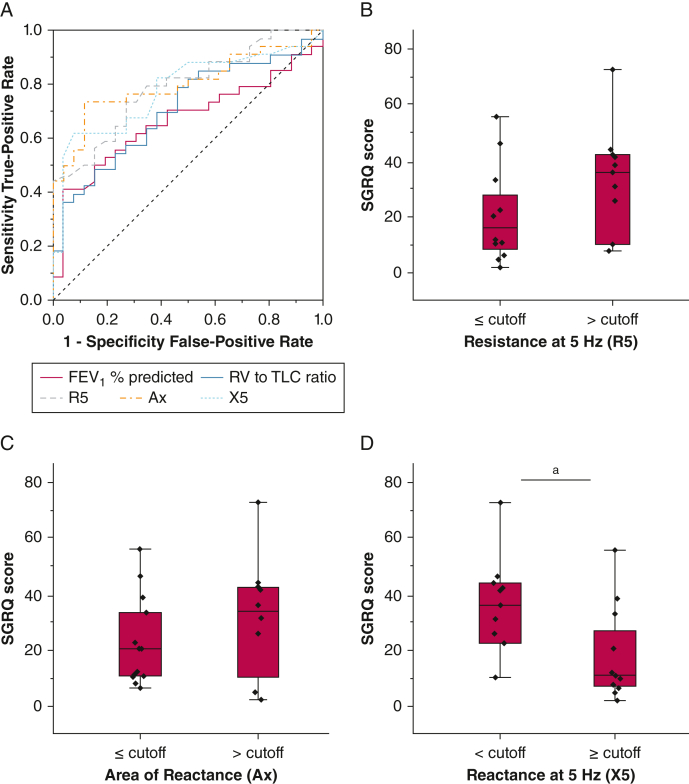
Receiver operating characteristic (ROC) curve analysis to predict worse SGRQ score and performance in a subgroup of patients with no functional pattern in lung function tests. A, ROC analysis revealed that oscillometry had the best performance to predict an SGRQ score of > 25, which signifies a high burden in quality of life (QoL), with Ax, R5, and X5 showing an area under the receiver operating characteristic curve (AUC) of 0.80, 0.79, and 0.78, respectively. From the conventional lung function tests, RV to TLC ratio and FEV_1_ % predicted showed the greater AUC (0.71 and 0.67, respectively). Interestingly, applying the cutoff values found in the ROC analysis in the subgroup of patients with neither an obstructive nor a restrictive functional pattern, X5 was the only oscillometry parameter that could identify the patients with worse QoL measure (D), in contrast to R5 (B) or Ax (C). Note that a more negative X5 (below cutoff) means worse reactance. Data are presented as median (IQR). ^a^*P* < .05. Ax = reactance area under the curve; R5 = resistance at 5 Hz; RV = residual volume; SGRQ = St. George’s Respiratory Questionnaire; TLC = total lung capacity; X5 = reactance at 5 Hz.

Both R5 and X5 were associated significantly with the total SGRQ score (ρ = 0.43 [95% CI, 0.19-0.62; *P* < .01] and ρ = –0.42 [95% CI, –0.61 to –0.18; *P* < .01], respectively) (Figure 2, Figure 3) and each of the individual SGRQ domain scores (Fig 3). R5-R20 and Ax showed a significant moderate correlation with SGRQ score (ρ = 0.35 [95% CI, 0.10-0.56] and 0.44 [95% CI, 0.20-0.63], respectively; *P* < .01 for both), suggesting that peripheral lung dysfunction was associated with worsening health status. In contrast, R20 showed only a weak relationship with the total SGRQ score (ρ = 0.26 [95% CI, 0.00-0.49]; *P* = .042). ROC curve analysis revealed that Ax best predicted the presence of an SGRQ score of > 25 (area under the receiver operating characteristic curve [AUC], 0.80; *P* < .001), followed by R5, X5, Fres, and R5-R20 (AUC, 0.79 [*P* < .001]; AUC, 0.78 [*P* < .001]; AUC, 0.76 [*P* < .001]; and AUC, 0.73 [*P* = .003]; respectively) (Fig 4, e-Table 2).

**e-Figure S1:**
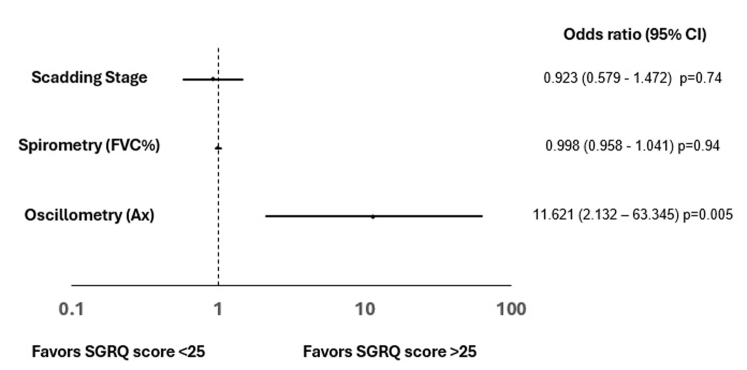


Most of the conventional lung function parameters (FEV_1_, TLCO, and TLC), showed no relationship with the total SGRQ score, although an association, albeit weak, was observed between FVC and SGRQ score (ρ = –0.30 [95% CI, –0.52 to –0.04]; *P* = .02) (Figure 2, Figure 3). TLCO, FEV_1_, and CPI were correlated weakly only with the impact domain of the SGRQ (ρ = –0.27 [95% CI, –0.49 to –0.01; *P* = .04]; ρ = –0.26 [95% CI, –0.54 to –0.07; *P* = .04]; and ρ = 0.29 [95% CI, 0.03-0.51; *P* = .03]; respectively). We did observe that the RV to TLC ratio, an indirect marker of gas trapping and peripheral airway dysfunction, was the only marker of lung volume that showed a weak but significant association with total SGRQ score (ρ = 0.30 [95% CI, 0.04-0.52] *P* = .02) (Figure 2, Figure 3). Interestingly, when Ax (oscillometry), FVC (spirometry), and the Scadding stage were included in a multivariable logistic regression, only Ax significantly predicted an increased SGRQ score (OR, 11.621 [95% CI, 2.132-63.345]; *P* = .005), whereas FVC (spirometry; *P* = .94) and Scadding stage (*P* = .74) did not (e-Fig 1). No connection was detected between Feno parameters and total SGRQ score or any domain.

### Hierarchy Cluster Analysis

**e-Figure S2:**
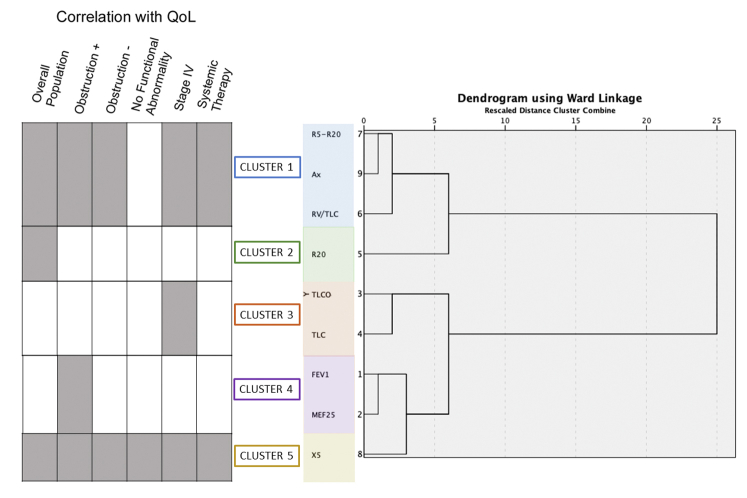


Lung function parameters were grouped by hierarchy cluster analysis into the following clusters: cluster 1, R5-R20, Ax, and RV to TLC ratio; cluster 2, R20; cluster 3, TLCO and TLC; cluster 4, FEV_1_ and MEF25%; and cluster 5, X5 (e-Fig 2). Although a detailed structure-function analysis cannot be performed in our study, some striking observations were noted. Cluster 1 includes R5-R20 and Ax, markers of peripheral airway involvement, as well as the RV to TLC ratio. Cluster 2 consists of R20, a marker of central airway obstruction. Cluster 3 comprises TLCO and TLC, both markers of restriction and alveolar-capillary membrane abnormalities. Cluster 4 includes FEV_1_ and MEF25%, both nonspecific markers of ventilatory abnormalities. Finally, X5 constituted an independent cluster.

## Discussion

In patients with sarcoidosis, oscillometry measurements of lung mechanics consistently are associated more strongly with QoL compared with conventional lung function tests. The most striking finding of our study was that peripheral lung oscillometry indices were related significantly to poor QoL measure. Specifically, R5, X5, Fres, AX, and R5-R20 were correlated with the total SGRQ scores and with each SGRQ domain score. ROC curve and regression analyses showed that oscillometry markers of peripheral lung dysfunction (X5, Ax, Fres, and R5-R20) and total airway resistance (R5) best predicted the presence of impaired respiratory QoL, outperforming spirometry.

The association of SGRQ score with peripheral lung function seen in our study agrees with studies of other respiratory diseases, such as COPD, where a better correlation of R5-R20 with SGRQ score was found compared with FEV_1_ % predicted.^28^ Our data suggest that oscillometry is more sensitive in correlating lung function with a QoL measure in sarcoidosis than conventional lung function techniques.

Sarcoidosis is a complex disease with both constitutional and organ-specific manifestations, resulting in impaired QoL as a result of multiple factors, for example, symptoms (fatigue, fever, cough, dyspnea), psychological impairment, physiologic disability, and comorbidities.^29^ Assessment of QoL is an important outcome for clinical trials in sarcoidosis^30^ and affects treatment decisions in everyday clinical practice.^4^ Common indices of lung function testing (ie, spirometry, body plethysmography, and gas diffusion) have been associated variably with QoL, with contradictory findings. In 200 patients with sarcoidosis, FEV_1_ % predicted, TLC % predicted, and TLCO correlated significantly (albeit not strongly) with the lung domain score of the King Sarcoidosis Questionnaire.^31^ In contrast, Cox et al^5^ reported no correlation of FEV_1_ % predicted with QoL, as assessed by either the 36-item Short Form Health Survey or the SGRQ score. According to our data, only FVC % predicted and RV to TLC ratio were associated weakly with the total SGRQ score, among the conventional lung function indices.

Our study is the first, to our knowledge, to report a significant relationship of oscillometry with SGRQ score in patients with sarcoidosis. Previously, Bade et al^32^ showed that both R5-R20 and X5 were higher in 28 patients with sarcoidosis compared with control participants and had a moderate correlation to FEV_1_ % predicted, in accordance with the relationship of FEV_1_ % predicted and oscillometry observed in our study. As expected, TLC % predicted was associated positively mainly with X5 and, to a lesser degree, associated negatively with Ax, but not with resistance. Previously, Suzuki et al^33^ also showed a significant correlation of vital capacity with X5 in patients with sarcoidosis. In patients with interstitial lung diseases, van Noord et al^34^ showed that TLC of < 80% predicted had more negative reactance at low frequencies compared with TLC of > 80% predicted.

Frequency dependence of both resistance (R5-R20) and reactance (Ax) are attributed to peripheral airway dysfunction, although tissue resistance and heterogeneities can increase R5-R20 and Ax.^6^ It is interesting to note the RV to TLC ratio, an indirect marker of peripheral airway disease, was associated significantly with R5-R20 and Ax, supporting the presence of small airways disease. Indeed, RV to TLC ratio is widely considered a measure of obstruction in the presence of restriction, where a mixture of past and present granulomatous small airways inflammation gives rise to combined obstruction and restriction. Previously, Faria et al^11^ also observed a significant association between oscillometric frequency dependence of resistance and RV to TLC ratio in patients with sarcoidosis.

The pathogenesis of small airways obstruction in sarcoidosis is multifactorial, including bronchiole obstruction by bronchiolar or peribronchial granulomas and fibrosis as well as elements of granulomatous bronchiolitis.^26^^,^^35^ Thus, it is possible that granulomatous infiltration around the small airways might be linked to impaired respiratory QoL. Proximal airway obstruction and the presence of traction bronchiectasis also may contribute to an obstructive pattern.^3^ Although the described prevalence of an obstructive pattern is broad among studies (4%-67%), it is associated with worse symptoms and increased morbidity.^36^ However, a favorable connection with mortality compared with mixed or restrictive ventilatory patterns also has been reported.^2^

Stratification of patients with sarcoidosis based on the presence or absence of airway obstruction did not reveal any differences between SGRQ scores among the groups (e-Table 3). Patients with an obstructive pattern showed the same value of oscillometric parameters of peripheral airway dysfunction (R5-R20 and Ax), suggesting that oscillometry is affected before spirometry in sarcoidosis and may detect small airways involvement more frequently compared with applying spirometric criteria of airway obstruction. Oscillometry parameters (excluding R20) were correlated significantly with the SGRQ score, regardless of the subgroup (obstructive vs nonobstructive), although stronger correlations were observed in the group of patients with obstructive spirometry findings (e-Tables 4, 5). Interestingly, FEV_1_ % predicted and MEF25% correlated with QoL only in the obstructive group.

In the subgroup of patients who did not meet criteria for either an obstructive or restrictive functional pattern, X5 was the only functional parameter significantly inversely correlated with SGRQ score (e-Tables 6, 7). Because reactance reflects the elastic properties of the lung tissue,^6^ it is possible that X5 may detect restrictive lung function abnormalities before alterations in TLC. Indeed, in a previous study using pressure-volume curves, 46.4% of symptomatic patients with elevated elastance showed normal TLC.^37^ R5 also may reflect alterations in peripheral airways, causing airflow limitation and uneven ventilation, despite nonobstructive spirometry findings.^38^ In total, X5 correlated with SGRQ score in the overall population and each subgroup analysis performed in our study, whereas it formed an independent factor in cluster analysis, emphasizing the importance of X5 as a global and sensitive measurement in sarcoidosis. We believe that this reflects the combined contribution of fibrotic changes and peripheral airway disease or heterogeneities in the effective elastance, that is, X5. Thus, oscillometric measures might well provide the most sensitive marker of the major burden of past (scar tissue) and current (inflammation) granulomatous disease activity in the lungs. However, the increased sensitivity of oscillometry (57.68% of the patients showed an abnormal R5-R20, in contrast to 37.10% with FEV_1_ < 80% predicted) raises the possibility that oscillometry has a greater signal to noise ratio, resulting in a better correlation with SGRQ score. Although for a more severe obstructive functional state, our subgroup analysis showed that oscillometry correlated significantly with SGRQ score, whether this also is applicable for a more severe restrictive pattern cannot be derived by our study because of a low number of patients with significantly reduced TLC.

Our cohort included a variety of sarcoidosis radiographic stages, including patients with fibrosis on chest radiography (one-third of total) or no parenchymal involvement (approximately 40%) (e-Table 8). Although our study does not include data on high-resolution CT imaging and the Scadding radiographic staging has several limitations,^39^ it is still used widely and is easier to perform for monitoring purposes compared with CT imaging. In stage IV, significant correlations with SGRQ score were noted for oscillometry parameters, except for R20, but also for CPI and TLCO (e-Table 9), reflecting the effect of fibrotic changes.^2^

In contrast to the previous studies by Tanizawa et al^40^ and Suzuki et al,^33^ our study did not exclude patients receiving systemic therapy. Although, assessing the impact of treatment on our results is beyond the scope of this study, it is noted that oscillometry remains the only technique that was correlated to SGRQ score (with the exemption of R20) in the subgroup of patients who received any immunosuppressive therapy (e-Table 10). Moreover, in contrast to Suzuki et al^33^ and Faria et al,^11^ smoking history was not an exclusion criterion in our study. No difference was observed between ever vs never smoking status (e-Table 11), although most patients who had ever smoked in our study were individuals who formerly smoked without a heavy smoking history (e-Appendix 1).

We acknowledge that the number of participants in our study does not allow definite subgroup analyses; that would require larger observational studies. Finally, a limitation of our study is that our results were not corrected for multiple comparisons and the cluster analysis was prespecified to 5 clusters, based on the rationale presented in the methodology.

Our study is the first, to our knowledge, to assess the correlation of Feno with SGRQ score in sarcoidosis, a rational hypothesis given that inflammation is associated with systemic symptoms (eg, fatigue^41^) that worsen QoL. Contrary to our hypothesis, Feno was not associated with QoL. Our data are in accordance with previous studies that do not support a role of Feno in sarcoidosis. Previously, Ziora et al^16^ found no correlation of exhaled NO levels with disease activity in patients with sarcoidosis, despite increased levels compared with control participants, whereas Wilsher et al^15^ showed that exhaled NO did not correlate with lung function parameters in sarcoidosis and was not increased compared with that in control participants. However, we acknowledge that Feno was measured in 44 of 62 patients, most of whom already were receiving inhaled or systemic corticosteroids, which severely impact Feno levels.^11^ In contrast, fatigue, a great contributor to QoL loss in sarcoidosis, seldom is suppressed by steroids. Thus, although the burden of granulomatous inflammation in the lung would fit with QoL, corticosteroids may mask any link with current airway inflammation (NO vs QoL), but not a link between past and present granulomatous burden and QoL.

## Interpretation

We showed that oscillometry parameters, especially those associated with the peripheral lung, are correlated more strongly to SGRQ, a respiratory QoL measure in sarcoidosis, compared with conventional lung function tests. Thus, oscillometry adds further information regarding the pathophysiologic consequences of sarcoidosis and may improve the current assessment and monitoring of the progression of sarcoidosis.

## Funding/Support

D. T. is supported by a long-term research fellowship from the European Respiratory Society.

## Financial/Nonfinancial Disclosures

The authors have reported to *CHEST* the following: D. T. reports honoraria from Menarini, Chiesi, 10.13039/100004325AstraZeneca, and 10.13039/100004330GlaxoSmithKline outside the submitted work. O. S. U. reports grants and personal fees from AstraZeneca, Boehringer Ingelheim, Chiesi, GlaxoSmithKline, Cipla, and Mereo Biopharma and honoraria from AstraZeneca, Boehringer Ingelheim, Chiesi, GlaxoSmithKline, Mundi Pharma, Sandoz, Takeda, Cipla, Covis, Novartis, Orion, Menarini, UCB, Trudell Medical, Deva, and Kamada outside the submitted work. None declared (K. K., P. P., A. B., M. B., H. K. L., H. K., C. M., N. D., G. A. K., P. J. B., A. U. W., E. A. R.).

## Supplementary Data

e-Online Data

e-Online Data
