# Supplementary material for: Contribution of Peripheral Airways Dysfunction to Poor Quality of Life in Sarcoidosis
Source: Chest. 2025 Mar 11;168(2):423–34. doi: 10.1016/j.chest.2025.02.036 (PMC12405914; doi:10.1016/j.chest.2025.02.036)
Supplement: e-Online Data [file mmc1.docx]

**E-FIGURE LEGENDS**

**E-FIGURE 1**. A multivariable logistic regression was performed to assess the effect of spirometry, oscillometry and Scadding stage to the SGRQ score. For the SGRQ score, a threshold value of 25 was used, previously proposed to indicate increased burden in QoL 7. From each lung function technique, the physiological parameter with the strongest correlation to the SGRQ in the univariate analysis, was chosen for the multivariable logistic regression analysis i.e., Ax from oscillometry and FVC% from spirometry. The overall model was statistically significant (chi-square = 20.836, p<0.001), explained 39.4% of the variation of SGRQ (Nagelkerke R2) and correctly predicted 73.3% of patients with increased respiratory QoL burden. Confirming our hypothesis, the oscillometric parameter Ax was the only variable that remained significantly associated with SGRQ>25 score on multivariable analysis [odds ratio 11.621 (2.132-63.345), p=0.005], whereas FVC% (spirometry) (p=0.94) and Scadding stage (p=0.74) were not.

**E-FIGURE 2**. Clustering of lung function parameters and correlation with SGRQ. Right, Dendrogram presenting the combination of the lung function parameters into the different clusters. Left, X5 that forms cluster 5, was the only parameter that correlated with the SGRQ of the overall population, as well as of each subgroup analyzed. Parameters of cluster 1 (R5-R20, Ax, RV/TLC ratio) were correlated with all groups, except for the subgroup with no functional abnormality. Interestingly, parameters of cluster 3 that characterize lung periphery correlated with SGRQ only in the group of patients at Stage IV, whereas parameters of cluster 4 correlated with SGRQ only in the group of patients with obstructive spirometric pattern. Grey color denotes a significant correlation with SGRQ of at least one parameter from the cluster.
