# Supplementary material for: Contribution of Peripheral Airways Dysfunction to Poor Quality of Life in Sarcoidosis
Source: Chest. 2025 Mar 11;168(2):423–34. doi: 10.1016/j.chest.2025.02.036 (PMC12405914; doi:10.1016/j.chest.2025.02.036)
Supplement: e-Online Data [file mmc2.docx]

**On-line data Supplement**

**Peripheral airways dysfunction is a major contributor to poor quality of life in sarcoidosis.**

Dimitrios Toumpanakis, Konstantinos Karagiannis, Paolo Paredi, Andras Bikov, Martina Bonifazi, Harpreet K Lota, Harpal Kalsi, Cosetta Minelli, Nikolaos Dikaios, George A Kastis, Peter J Barnes, Athol U Wells, Omar S Usmani, Elisabetta A Renzoni A

**METHODS**

**Impulse oscillometry**

Oscillometry measurements were performed using a Jaeger Master Screen Impulse Oscillometry system (Jaeger Co, Wurzburg, Germany), as previously described by our group ^1^. Briefly, three reproducible sessions each of 60-seconds were recorded, and maneuvers with artefacts caused by coughing, breath-holding, swallowing, or vocalization were excluded ^2^. Oscillometry uses the superimposition of pressure waves applied to the mouth during tidal breathing to determine resistance (R) and reactance (X, the net effect of elastance and inertance) across the frequency range ^3^. Reported variables are resistance at 5 and 20 Hz (R5, R20, respectively), frequency dependence of resistance (R5-R20), reactance at 5 Hz (X5), area under the reactance curve (Ax) and resonant frequency (Fres). The average value from three acceptable measurements is reported.

R5 and R20 represent total and central airway resistance, respectively, thus their difference (R5-R20, frequency dependence of resistance) is used as a marker of peripheral airway dysfunction, although it can also be affected by other sources of ventilation inhomogeneity ^4^. X5 is the reactance at 5 Hz and Ax is the reactance area under the curve i.e., frequency dependence of reactance. Although both are affected by changes in tissue elastance, it is increasingly shown the X5 and Ax are also sensitive to small airway disease. Finally, Fres is the resonant frequency where elastance equals inertance that results in zero reactance.

**Hierarchy Cluster Analysis**

Hierarchy cluster analysis (HCA) based on the Ward’s minimum variance method ^5^ and the Squared Euclidean Distance as the similarity measure was performed to classify the evaluated parameters into 5 clusters. HCA was performed using the SPSS software. The Ward method instead of optimizing the distances between clusters, it commonly uses the sum of squared deviations (or other criteria) from the mean of a cluster to optimize the clusters homogeneity. The advantage of this method is that it (i) tends to avoid small clusters and (ii) minimizes intra-cluster dispersion. Since each variable is in different units and different scales, they were standardized using the Z-Score formula.

Analysis was prespecified to 5 clusters, based on the common interpretation of oscillation mechanics to 3 compartments i.e., central airways, peripheral airways and lung tissue elastance (3), adding also the effect of heterogeneities and a fifth group, for non-specific markers. The following lung function parameters were included in the analysis: FEV1%, MEF25% (spirometry), TLC%, RV/TLC (body plethysmography), TLCO% (single breath CO uptake) and R5-R20, R20, X5, Ax (oscillometry).

**Interpretation of correlation coefficient**

Interpretation of correlation coefficients, when statistically significant, followed the stratification suggested by Schober et al ^6^ depending on Rho numerical value, i.e. weak (Rho≥0.10-0.39), moderate (Rho≥0.40-0.69) and strong (Rho≥0.70) correlation.

**RESULTS**

**e-Table 1.** Correlation of lung function parameters assessed by oscillometry with exhaled nitric oxide.

|  | FE_NO_50 (ppb) | J’awNO (pL/sec) | C_A_NO (ppb) |
| --- | --- | --- | --- |
| R5 (kPa/L/s) | NS | -0.37 (-0.62,-0.04) (p=0.025) | NS |
| R20 (kPa/L/s) | NS | -0.37 (-0.62,-0.04) (p=0.026) | NS |
| R5-R20 (kPa/L/s) | NS | NS | NS |
| X5 (kPa/L/s) | NS | NS | NS |
| Fres (Hz) | NS | NS | NS |
| Ax (kPa/L) | NS | NS | NS |

Values are Spearman’s correlation coefficient – Rho (95% confidence intervals) (p value), NS = non statistically significant

**e-Table 2.** Receiver-operating characteristic (ROC) curve analysis of lung function parameters to predict impaired respiratory quality of life (QoL), as defined by a total SGRQ score>25.

|  | Area Under the Curve (AUC) | p-value | 95% LCL | 95% UCL | Cut-Off Value |
| --- | --- | --- | --- | --- | --- |
| FEV1% | 0.67 | 0.02 | 0.52 | 0.82 | 86.55 |
| FVC% | 0.65 | 0.04 | 0.51 | 0.80 | 99.10 |
| FEV1/FVC | 0.55 | 0.52 | 0.40 | 0.70 | 0.74 |
| MEF25% | 0.56 | 0.41 | 0.41 | 0.71 | 76.50 |
| TLCO% | 0.63 | 0.08 | 0.48 | 0.78 | 68.45 |
| RV/TLC ratio | 0.71 | 0.007 | 0.57 | 0.84 | 34.37 |
| TLC% | 0.59 | 0.22 | 0.44 | 0.74 | 93.65 |
| CPI | 0.61 | 0.14 | 0.46 | 0.76 | 21.56 |
| R5 (kPa/L/s) | 0.79 | <0.001 | 0.67 | 0.91 | 0.43 |
| R20 (kPa/L/s) | 0.69 | 0.01 | 0.56 | 0.83 | 0.35 |
| R5-R20 (kPa/L/s) | 0.73 | 0.003 | 0.59 | 0.86 | 0.09 |
| X5 (kPa/L/s) | 0.78 | <0.001 | 0.65 | 0.91 | -0.145 |
| Ax (kPa/L) | 0.80 | <0.001 | 0.68 | 0.92 | 0.66 |
| Fres (Hz) | 0.76 | <0.001 | 0.64 | 0.89 | 15.56 |

LCL, lower confidence limit, UCL, upper confidence limit.

**e-Figure 1.** A multivariable logistic regression was performed to assess the effect of spirometry, oscillometry and Scadding stage to the SGRQ score. For the SGRQ score, a threshold value of 25 was used, previously proposed to indicate increased burden in QoL ^7^. From each lung function technique, the physiological parameter with the strongest correlation to the SGRQ in the univariate analysis, was chosen for the multivariable logistic regression analysis i.e., Ax from oscillometry and FVC% from spirometry. The overall model was statistically significant (chi-square = 20.836, p<0.001), explained 39.4% of the variation of SGRQ (Nagelkerke R^2^) and correctly predicted 73.3% of patients with increased respiratory QoL burden. Confirming our hypothesis, the oscillometric parameter Ax was the only variable that remained significantly associated with SGRQ>25 score on multivariable analysis [odds ratio 11.621 (2.132-63.345), p=0.005], whereas FVC% (spirometry) (p=0.94) and Scadding stage (p=0.74) were not.


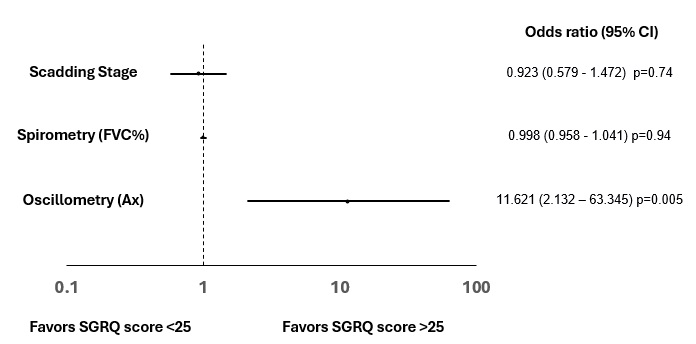


**e-Figure 2.** Clustering of lung function parameters and correlation with SGRQ. *Right,* Dendrogram presenting the combination of the lung function parameters into the different clusters. *Left*, X5 that forms cluster 5, was the only parameter that correlated with the SGRQ of the overall population, as well as of each subgroup analyzed. Parameters of cluster 1 (R5-R20, Ax, RV/TLC ratio) were correlated with all groups, except for the subgroup with no functional abnormality. Interestingly, parameters of cluster 3 that characterize lung periphery correlated with SGRQ only in the group of patients at Stage IV, whereas parameters of cluster 4 correlated with SGRQ only in the group of patients with obstructive spirometric pattern. Grey color denotes a significant correlation with SGRQ of at least one parameter from the cluster.


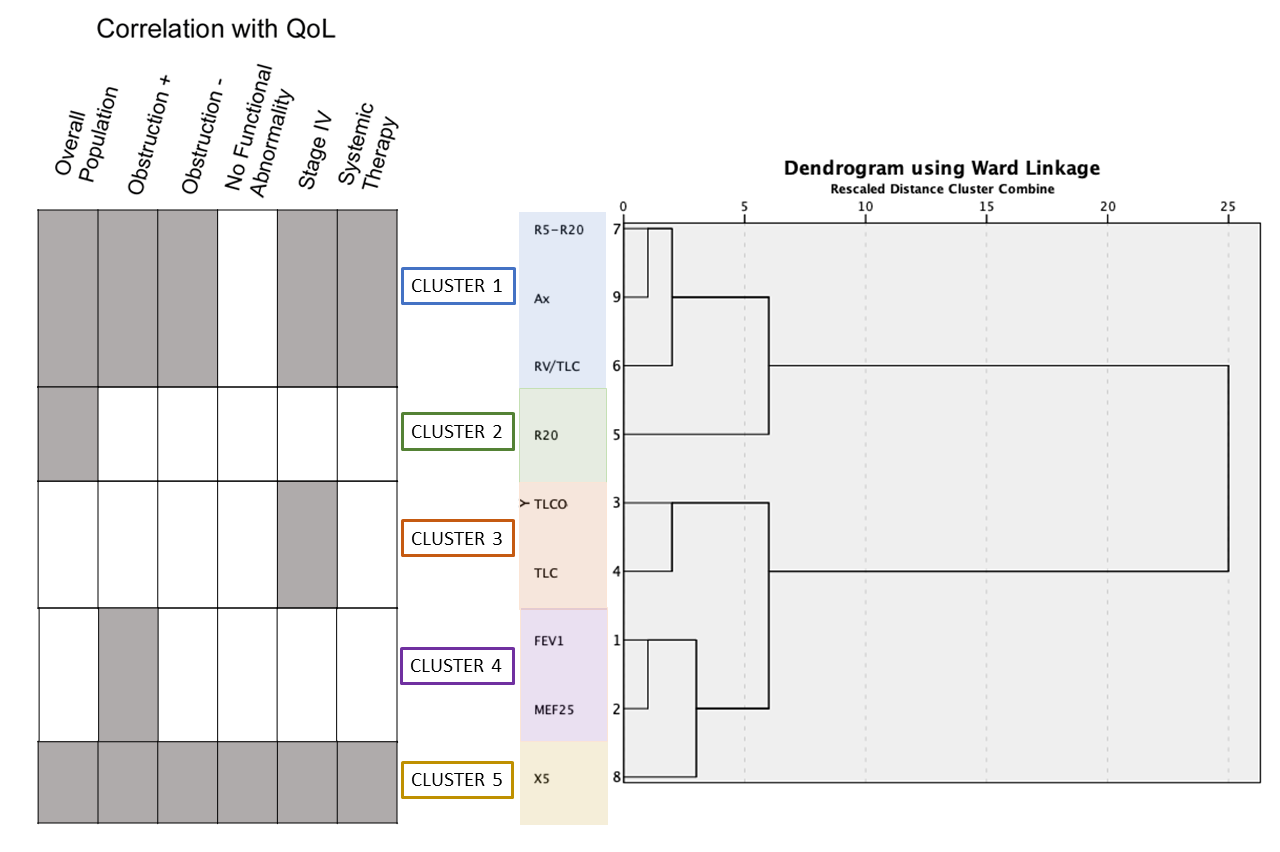


**Subgroup analysis based on the presence of spirometric obstructive pattern.**

In view of the known heterogeneity of ventilatory impairment patterns in sarcoidosis, patients were subdivided into two groups based on the presence, or absence of obstructive pattern on lung function tests (for baseline characteristics of the subgroups, see supplementary e-Table 3). Significant airway obstruction was defined as FEV_1_/FVC ratio less than 0.7. Using this criterion, 23 of our 62 patients (37.1%) had airway obstruction [FEV1/FVC 0.63 (0.53-0.68)] and 38 patients were non-obstructed [FEV_1_/FVC 0.80 (0.75—0.83)]. The correlation coefficients between lung function tests and SGRQ scores (total/domains) for obstructive and non-obstructive groups are summarized in e-Tables 5 and 6, respectively. In patients with airway obstruction, both oscillometry indices (except R20) and conventional lung function parameters linked to obstruction severity (FEV_1_%, MEF25%) and gas trapping (RV/TLC ratio) were significant associated with a higher questionnaire score (worst QoL), although R5-R20 failed to reach statistical significance [Rho= 0.37 (-0.07,0.68), p=0.08] (e-Table 4). The strongest correlation was observed for Fres and AX among oscillometry measurements [rho=0.55 (0.16,0.79), p<0.01 and rho=0.55 (0.17,0.79), p<0.01 to total SGRQ score, respectively]. The absolute value of FEV1/FVC ratio was not associated to total SGRQ. In patients without significant airway obstruction, again only distal oscillometry indices (R5, X5, AX, R5-R20) were significantly correlated with higher questionnaire scores, although Fres failed to reach statistical significance [Rho=0.29 (-0.05,0.57), p=0.08], whereas none of the conventional lung function parameters (e.g., FEV1%, FVC%, TLCO%, TLC%) were significantly linked (e-Table 5).

**e-Table 3.** Baseline characteristics of patients’ subgroups depending on the presence or not of an obstructive pattern in spirometry.

|  | Non-Obstructive Pattern  (n=39) | Obstructive Pattern  (n=23) |
| --- | --- | --- |
| Age (y) | 50.0 (42-58) | 59 (49-65) * |
| Restrictive Pattern | 11 (28.2%) | 5 (21.7%) |
| CPI | 26.8 (15.4-42.6) | 18.44 (4.27-27.3) * |
| FEV1% | 90.7 (77.3-102.5) | 73.7 (60.7-88.8) * |
| FVC% | 96.3 (79.2-108.3) | 103.0 (81.0-109.4) |
| FEV1/FVC | 0.80 (0.75—0.83) | 0.63 (0.53-0.68) * |
| TLC% | 88.0 (78.4-98.3) | 98.5 (84.9-107.0) * |
| RV/TLC ratio | 32.6 (29.3-37.2) | 36.5 (29.3-43.4) |
| TLCO% | 71.5 (50.2-80.2) | 66.9 (63.1-86.4) |
| KCO% | 88.0 (79.3-96.3) | 87.2 (72.7-107.0) |
| MEF25% | 46.8 (33.3-60.8) | 22.1 (16.5-31.9) * |
| FE_NO_50 (ppb) | 20.9 (13.4-26.4) | 21.5 (14.6-30.6) |
| J’awNO (pL/sec) | 925.2 (585.9-1068.0) | 907.0 (601.7-1280.5) |
| C_A_NO (ppb) | 4.24 (2.95-5.94) | 3.23 (2.08-5.20) |
| R5 (kPa/L/s) | 0.45 (0.37-0.54) | 0.43 (0.34-0.58) |
| R20 (kPa/L/s) | 0.35 (0.30-0.43) | 0.36 (0.29-0.41) |
| R5-R20 (kPa/L/s) | 0.10 (0.05-0.13) | 0.08 (0.04-0.22) |
| X5 (kPa/L/s) | -0.15 [-0.20-(-0.12)] | -0.14 [-0.24-(-0.12)] |
| Fres (Hz) | 15.36 (12.63-19.11) | 16.5 (13.3-22.07) |
| Ax (kPa/L) | 0.75 (0.36-1.08) | 0.60 (0.27-2.36) |
| Total SGRQ | 31.29 (11.78-43.77) | 25.16 (14.86-46.18) |
| Symptoms domain | 33.22 (10.78-51.00) | 37.21 (14.78-54.01) |
| Activity domain | 47.66 (24.31-66.19) | 30.61 (11.21-59.80) |
| Impact domain | 24.21 (5.60-42.57) | 16.45 (7.50-34.17) |

Data presented as Median (IQR), * p<0.05 to non-obstructive group.

**e-Table 4.** Correlations in patients with presence of obstruction (N=23)

|  | SGRQ | | | |
| --- | --- | --- | --- | --- |
| Lung Function Parameter | Total | Symptoms | Activity | Impact |
| R5 (kPa/L/s) | 0.45 (0.04,0.74 (p=0.03) | 0.50 (0.10,0.76) (p=0.02) | NS | 0.46 (0.05,0.74) (p=0.02) |
| R20 (kPa/L/s) | NS | NS | NS | NS |
| R5 – R20 (kPa/L/s) | NS | 0.44 (0.03,0.73) (p=0.03) | NS | NS |
| X5 (kPa/L/s) | -0.48 (-0.75,-0.07) (p=0.02) | -0.47 (-0.75,-0.07) (0.02) | NS | -0.49 (-0.76,-0.09) (p=0.02) |
| Fres (Hz) | 0.55 (0.16,0.79) (p<0.01) | 0.54 (0.15,0.78) (p<0.01) | 0.44 (0.02,0.73) (p=0.03) | 0.47 (0.06,0.75) (p=0.02) |
| Ax (kPa/L) | 0.55 (0.17,0.79) (p< 0.01) | 0.55 (0.16,0.79) (p<0.01) | 0.43 (0.01,0.72) (0.04) | 0.49 (0.08,0.75) (p=0.02) |
| FEV1% | -0.48 (-0.75,-0.07) (p=0.02) | -0.54 (-0.79,-0.16) (p<0.01) | NS | NS |
| FVC% | NS | NS | NS | NS |
| FEV1/FVC | NS | -0.46 (-0.74,-0.05) (p=0.03) | NS | NS |
| TLCO% | NS | NS | NS | NS |
| KCO% | NS | NS | NS | NS |
| TLC% | NS | NS | NS | NS |
| CPI | NS | NS | NS | NS |
| RV/TLC ratio | 0.50 (0.09,0.76) (p=0.01) | 0.57 (0.20,0.80) (p<0.01) | NS | 0.44 (0.02,0.73) (p=0.04) |
| MEF25% | -0.49 (-0.76,-0.07) (p=0.02) | -0.60 (-0.82,-0.23) (p<0.01) | NS | NS |

Values are Spearman’s correlation coefficient – Rho (95% confidence intervals) (p value), NS = non statistically significant

**e-Table 5.** Correlations in patients with absence of obstruction (N=39)

|  | SGRQ | | | |
| --- | --- | --- | --- | --- |
| Lung Function Parameter | Total | Symptoms | Activity | Impact |
| R5 (kPa/L/s) | 0.38 (0.06,0.64) (p=0.02) | 0.35 (0.14,0.61)  (p=0.04) | 0.37 (0.04,0.63) (p=0.02) | 0.40 (0.08,0.65) (p=0.01) |
| R20 (kPa/L/s) | NS | NS | NS | NS |
| R5 – R20 (kPa/L/s) | 0.33 (0.00,0.60) (p=0.04) | 0.37 (0.04,0.62) (p=0.02) | NS | 0.42 (0.10,0.66) (p<0.01) |
| X5 (kPa/L/s) | -0.37 (-0.62,-0.04) (p=0.03) | -0.33 (-0.60,-0.00) (p=0.04) | NS | -0.36 (-0.62,-0.03) (p=0.03) |
| Fres (Hz) | NS | NS | NS | NS |
| Ax (kPa/L) | 0.38 (0.05,0.63) (p=0.02) | 0.34 (0.00,0.60) (p=0.04) | NS | 0.40 (0.09,0.65) (p=0.01) |
| FEV1% | NS | NS | NS | NS |
| FVC% | NS | NS | NS | NS |
| FEV1/FVC | NS | NS | NS | NS |
| TLCO% | NS | NS | NS | NS |
| KCO% | NS | NS | NS | NS |
| TLC% | NS | NS | NS | NS |
| CPI | NS | NS | NS | NS |
| RV/TLC ratio | NS | NS | NS | NS |
| MEF25% | NS | NS | NS | NS |

Values are Spearman’s correlation coefficient – Rho (95% confidence intervals) (p value), NS = non statistically significant

**Subgroup analysis based on the absence of either an obstructive or a restrictive pattern in spirometry and body plethysmography.**

A separate analysis was performed in 23 patients with neither an obstructive nor a restrictive pattern in spirometry and body plethysmography. Patients with a smoking history of >10 pack.years were also excluded to prevent a possible confounding contribution of a significant previous smoking exposure. As seen in supplementary e-Table 6, X5 was the only lung function parameter with a significant moderate negative correlation with SGRQ [Rho=-0.49 (-0.75,-0.08), p=0.02]. Indeed, applying in this subgroup, the cut-off values of the parameters with a statistically significant ROC curve analysis in the overall population, X5 was again the only factor that discriminated between better and worse respiratory QoL (e-table 7)

**e-Table 6**. Lung function parameters and correlation with total SGRQ score.

| Lung Function Parameter | | Correlation with QoL |
| --- | --- | --- |
| CPI | 19.06 (12.43-29) | NS |
| FEV1% | 95.6 (87.5-105) | NS |
| FVC% | 101.9 (90.7-1088) | NS |
| FEV1/FVC | 0.79 (0.75-0.82) | NS |
| TLCO% | 77.1 (63.3-81.7) | NS |
| KCO% | 90.6 (76.8-96.7) | NS |
| RV% | 90.3 (79.4-104.8) | NS |
| TLC% | 94.2 (87.6-100.4) | NS |
| RV/TLC ratio | 31.85 (28.43-35.08) | NS |
| MEF25% | 50.6 (33.3-62.7) | NS |
| R5 | 0.41 (0.35-0.54) | NS |
| R20 | 0.35 (0.31-0.44) | NS |
| R5-R20 | 0.08 (0.04-0.11) | NS |
| X5 | -0.15 [-0.19-(-0.11)] | -0.49 (-0.75,-0.08) ***** |
| Fres | 15.29 (11.75-17.77) | NS |
| Ax | 0.58 (0.32-0.87) | NS |

Data presented as median (IQR) and Rho value (95% confidence intervals). *p<0.05, NS = non statistically significant

**e-Table 7**. SGRQ score in patients with non-functional spirometric abnormality, using the cut off values estimated by the ROC curve analysis in the overall population.

| Lung Function Parameter with significant ROC curve analysis | Total SGRQ Score | |
| --- | --- | --- |
|  | below cut-off | above cut-off |
| FEV1% | 28.43 (10.44-42.27) | 20.46 (10.11-38.45) |
| FVC% | 25.78 (10.44-42.27) | 21.43 (10.11-38.45) |
| RV/TLC ratio | 20.41 (10.11-38.45) | 28.43 (10.33-41.84) |
| R5 | 16.07 (8.28-27.73) | 35.90 (10.11-42.27) |
| R5-R20 | 20.46 (10.73-33.06) | 33.49 (7.73-42.27) |
| X5 | 35.90 (22.40-43.77) | 11.26 (6.93-26.76) * |
| Fres | 20.46 (9.93-38.45) | 33.49 (10.27-43.02) |
| Ax | 20.36 (10.44-33.06) | 33.49 (10.11-42.27) |

Data presented as median (IQR), *p<0.05

**Subgroup Analysis based on the Scadding imaging stage.**

A further subgroup analysis was performed based on chest imaging and patients were classified into 2 groups, i.e., stage IV (presence of fibrotic changes) versus other Scadding stages (pooled). Patient characteristics and correlations of lung function parameters with SGRQ are shown in e-Tables 8 and 9, respectively.

There was an increased proportion of restrictive functional pattern (47%) in patients in stage IV, compared to other groups (16%), with more severely affected lung function, e.g. reduced FEV1%, TLCO% and FVC%. Regarding oscillometry, only X5 was worse in stage IV (more negative), whereas no difference was observed in R5-R20 or Ax. Despite these differences, patients had the same impact on QoL, as assessed by the total SGRQ questionnaire. Interestingly, patients in different imaging stages had differential correlation of SGRQ with lung function indices, as shown in e-Table 8. For patients in stage IV, significant correlations with SGRQ were noticed for CPI [rho=0.54 (0.08,0.81), p=0.02], TLCO% [rho=-0.60 (-0.84,-0.17), p=0.01] and oscillometry parameters, except for R20, with the strongest correlation seen for R5-R20 [rho=0.59 (0.15,0.83), p=0.01]. For patients on all other stages (pooled), only oscillometry correlated with SGRQ, with the exemption of R5-R20.

**e-Table 8.** Subgroup analysis of our cohort based on radiographic staging.

|  | Other stages (N=43) | Stage IV (N=19) |
| --- | --- | --- |
| Age (y) | 54 (44-63) | 52.0 (27.0-64.0) |
| BMI (kg.m^-2^) | 28.07 (25.7-33.57) | 23.0 (22.2-27.7) * |
| Ever Smoking | 17 (40%) | 5 (26%) ^ |
| Obstructive Pattern | 14 (32.5%) | 9 (47.4%) |
| Restrictive Pattern | 7 (16.3%) | 9 (47.4%) ^ |
| CPI | 19.05 (10.99-26.82) | 38.9 (27.3-47.6) * |
| FEV1% | 90.7 (77.3-101.8) | 70.1 (59.1-80.0) * |
| FVC% | 100.3 (88.2-109.4) | 84.7 (64.3-106.9) * |
| FEV1/FVC | 0.75 (0.68-0.80) | 0.71 (0.61-0.82) |
| TLCO% | 78.2 (66.7-83.8) | 50.4 (38.9-63.3) * |
| KCO% | 93.1 (81.8-100.6) | 75.5 (64.8-85.3) * |
| RV% | 93.6 (79.4-109.3) | 86.6 (69.1-103.3) |
| TLC% | 94.9 (84.9-102.8) | 80.8 (70.3-100.6) |
| RV/TLC ratio | 32.5 (28.4-37.3) | 36.5 (32.6-42.1) * |
| MEF25% | 42.3 (26.0-57.4) | 30.3 (21.9-42.1) |
| FE_NO_50 (ppb) | 21.5 (13.6-29.0) | 18.5 (13.7-25.8) |
| J’awNO (pL/sec) | 913.5 (589.4-1280.5) | 1007.2 (610.4-1068.0) |
| C_A_NO (ppb) | 3.2 (2.4-5.4) | 5.94 (3.36-5.99) |
| R5 | 0.43 (0.34-0.54) | 0.45 (0.39-0.54) |
| R20 | 0.35 (0.29-0.43) | 0.34 (0.30-0.39) |
| R5-R20 | 0.10 (0.04-0.12) | 0.11 (0.05-0.16) |
| X5 | -0.14 [-0.17-(-0.10)] | -0.19 [-0.28-(-0.14)] * |
| Fres | 15.3 (12.6-19.2) | 16.5 (13.8-21.8) |
| Ax | 0.71 (0.26-1.04) | 0.81 (0.50-1.62) |
| Total SGRQ score | 30.9 (11.8-42.2) | 30.2 (15.3-46.3) |
| Symptom domain | 31.8 (10.7-47.6) | 41.5 (18.5-54.0) |
| Activity domain | 43.2 (17.4-65.6) | 30.1 (24.3-66.2) |
| Impact domain | 19.3 (5.6-32.4) | 24.7 (7.7-43.0) |

Data presented as Median (IQR), * p<0.05, ^ p<0.05 chi-squared test.

**e-Table 9.** Subgroup analysis of our cohort based on radiographic staging and correlation with SGRQ.

|  | Other Stages | Stage IV |
| --- | --- | --- |
| CPI | NS | 0.54 (0.08,0.81) ***** |
| FEV1% | NS | NS |
| FVC% | NS | NS |
| FEV1/FVC | NS | NS |
| TLCO% | NS | -0.60 (-0.84,-0.17) ***** |
| KCO% | NS | NS |
| RV% | NS | NS |
| TLC% | NS | NS |
| RV/TLC ratio | NS | NS |
| MEF25% | NS | NS |
| R5 | 0.40 (0.11,0.64) ***** | 0.47 (-0.01,0.77) ***** |
| R20 | 0.35 (0.04,0.60) ***** | NS |
| R5-R20 | NS | 0.59 (0.15,0.83) ***** |
| X5 | -0.36 (-0.60,-0.05) ***** | -0.53 (-0.80,-0.06) ***** |
| Fres | 0.34 (0.03,0.59) ***** | 0.51 (0.04,0.79) ***** |
| Ax | 0.36 (0.05,0.61) ***** | 0.54 (0.08,0.81) ***** |

Values are Spearman’s correlation coefficient – Rho (95% confidence intervals) (p value), NS = non statistically significant

**Subgroup Analysis in patients receiving systemic immunosuppressive therapy.**

Our study was a real-life observational study including patients already on treatment. As described in main text, 11 (17.7%) of our patients did not receive any treatment for sarcoidosis, 5 (8%) received only inhaled corticosteroids (ICS) and 46 (74.2%) received systemic immunosuppressive treatment. In accordance with the results in the overall population, oscillometry was the only functional technique that corelated with SGRQ in patients on systemic therapy (e-Table 10), with Ax having the stronger correlation (rho=0.47, p<0.01). Resistance of the large airways (R20) showed no significant correlation with SGRQ.

**e-Table 10.** Baseline characteristics of the subgroup of patients under systemic immunosuppressive therapy and correlation of lung function and FE_NO_50 with total SGRQ score.

|  | Any systemic immunosuppressive therapy (n=46) | Correlation of lung function parameters with total SGRQ score |
| --- | --- | --- |
| Sex (F/M) | 21/25 |  |
| Obstructive Pattern | 37% |  |
| Restrictive Pattern | 32.61% |  |
| Age (y) | 52 (44-63) |  |
| FEV1% pred | 81.45 (63.7-92.2) | NS |
| FVC % pred | 89.5 (78.5-104.0) | NS |
| FEV1/FVC | 0.74 (0.67-0.81) | NS |
| TLco% pred | 64.7 (47.4-78.9) | NS |
| Kco % pred | 85.25 (72.9-98.3) | NS |
| TLC% pred | 86.4 (77.9-97.9) | NS |
| RV/TLC ratio | 34.77 (29.39-40.74) | NS |
| MEF25% pred | 34.7 (24.6-48.7) | NS |
| CPI | 27.37 (18.24-39.34) | NS |
| R5 (kPa/L/s) | 0.45 (0.37-0.55) | 0.43 (0.14,0.65) * |
| R20 (kPa/L/s) | 0.36 (0.30-0.41) | NS |
| R5 - R20 (kPa/L/s) | 0.10 (0.05-0.14) | 0.42 (0.12,0.64) * |
| X5 (kPa/L/s) | -0.15 [-0.21-(-0.12)] | -0.36 (-0.60,-0.07) * |
| Fres (Hz) | 16.12 (13.8-20.62) | 0.45 (0.17,0.67) * |
| Ax (kPa/L) | 0.78 (0.40-1.37) | 0.47 (0.19,0.68) * |
| FE_NO_50 (ppb) | 22.25 (16.55-27.35) |  |
| C_A_NO (ppb) | 4.44 (2.87-5.97) |  |
| J’awNO (pL/sec) | 987.33 (655.34-1229.55) |  |
| Total SGRQ | 34.24 (16.8-51.12) |  |
| Symptoms Domain | 37.08 (9.88-53.49) |  |
| Activity Domain | 50.01 (29.49-72.93) |  |
| Impact Domain | 25.51 (7.61-42.78) |  |

Data presented as Median (IQR) (middle column) and Spearman’s correlation coefficient- Rho (95% confidence intervals) (right column). * p<0.05, NS = non statistically significant

**The effect of smoking history**

To evaluate the effect of smoking history on the peripheral lung function and SGRQ score of sarcoidosis patients, a subgroup analysis was performed between never smokers and ever (ex- or current) smokers. Our results revealed the absence of any significant effect of smoking history on the physiological features or SGRQ in our cohort (e-Table 11). Smoking exposure has conflicting results in sarcoidosis natural course, with studies suggesting both a protective effect ^8^ and a negative effect with increased prevalence of an obstructive pattern ^9^. Moreover, smoking may independently affect the lung, causing peripheral airway dysfunction, even in the absence of any changes in spirometry and can act as a confounding factor for small airway dysfunction in patients with sarcoidosis ^10^. Finally, the absence of any effect of smoking in sarcoidosis has also been reported ^11^, as in our cohort. However, it must be acknowledged that in our cohort, the vast majority of the “ever smokers” group (95.5%) were ex-smokers with a median time since smoking cessation of 20.5 y (10-30) and without a heavy cumulative exposure [4.5 pack.years (3-15)], while only one young occasionally active smoker was present.

**e-Table 11.** Effect of smoking history on lung function and SGRQ in sarcoidosis patients.

|  | Never smokers | Ever smokers |
| --- | --- | --- |
|  | n=40 | n=22 (current=1, ex=21)) |
| Age (y) | 53 (45.5-64) | 53.5 (45-63) |
| Sex (F/M) | 23/17 | 7/15 |
| Obstructive pattern | 13 (32.5%) | 10 (45.5%) |
| Restrictive pattern | 11 (28%) | 5 (23%) |
| FEV1% | 88.4 (64.85-99.15) | 79.25 (67.00-98.70) |
| FVC % | 98.05 (82.3-108.1) | 99.00 (81.00-110.50) |
| FEV1/FVC | 0.76 (0.68-0.81) | 0.73 (0.58-0.82) |
| TLCO% | 67.80 (53.85-79.60) | 72.55 (55.60-86.40) |
| KCO % | 87.00 (76.7-93.35) | 87.45 (72.70-100.60) |
| RV% | 87.70 (69.10-103.80) | 96.40 (85.00-113.50) |
| TLC% | 89.90 (78.40-100.40) | 96.50 (83.20-105.90) |
| RV/TLC ratio | 34.93 (29.28-41.25) | 33.52 (29.27-39.19) |
| MEF25% | 38.55 (25.3-51.35) | 42.10 (22.20-53.00) |
| CPI | 25.02 (13.19-38.33) | 19.92 (3.69-30.10) |
| R5 (kPa/L/s) | 0.45 (0.36-0.54) | 0.43 (0.37-0.55) |
| R20 (kPa/L/s) | 0.36 (0.29-0.40) | 0.35 (0.30-0.42) |
| R5 - R20 (kPa/L/s) | 0.10 (0.045-0.13) | 0.078 (0.05-0.14) |
| X5 (kPa/L/s) | -0.148 (-0.20-(-0.115)) | -0.1525 (-0.19-(-0.12)) |
| Fres (Hz) | 15.47 (12.26-19.15) | 16.91 (14.20-22.70) |
| Ax (kPa/L) | 0.663 (0.31-1.105) | 0.797 (0.36-1.50) |
| FE_NO_50 (ppb) | 18.50 (11.67-25.80) | 24.60 (15.78-31.05) |
| C_A_NO (ppb) | 882.16 (585.85-1068.00) | 982.29 (610.42-1347.50) |
| J’awNO (pL/sec) | 4.01 (2.09-5.96)) | 3.36 (2.85-5.49) |
| Total SGRQ | 29.34 (10.73-46.27) | 31.29 (21.57-40.64) |
| Symptoms Domain | 34.52 (8.98-51.68) | 36.94 (21.16-47.64) |
| Activity Domain | 35.60 (18.47-59.46) | 42.20 (17.43-66.19) |
| Impact Domain | 21.42 (5.60-39.09) | 19.55 (13.09-32.39) |

Data presented as Median (IQR)

**REFERENCES**

1. Paredi P, Goldman M, Alamen A, Ausin P, Usmani OS, Pride NB, Barnes PJ. Comparison of inspiratory and expiratory resistance and reactance in patients with asthma and chronic obstructive pulmonary disease. *Thorax* 2010; 65: 263-267.

2. Bikov A, Pride NB, Goldman MD, Hull JH, Horvath I, Barnes PJ, Usmani OS, Paredi P. Glottal Aperture and Buccal Airflow Leaks Critically Affect Forced Oscillometry Measurements. *Chest* 2015; 148: 731-738.

3. King GG, Bates J, Berger KI, Calverley P, de Melo PL, Dellaca RL, Farre R, Hall GL, Ioan I, Irvin CG, Kaczka DW, Kaminsky DA, Kurosawa H, Lombardi E, Maksym GN, Marchal F, Oppenheimer BW, Simpson SJ, Thamrin C, van den Berge M, Oostveen E. Technical standards for respiratory oscillometry. *Eur Respir J* 2020; 55.

4. Kaminsky DA, Simpson SJ, Berger KI, Calverley P, de Melo PL, Dandurand R, Dellaca RL, Farah CS, Farre R, Hall GL, Ioan I, Irvin CG, Kaczka DW, King GG, Kurosawa H, Lombardi E, Maksym GN, Marchal F, Oostveen E, Oppenheimer BW, Robinson PD, van den Berge M, Thamrin C. Clinical significance and applications of oscillometry. *Eur Respir Rev* 2022; 31.

5. Ward JH. Hierarchical Grouping to Optimize an Objective Function. *Journal of the American Statistical Association* 1963; 58: 236-244.

6. Schober P, Boer C, Schwarte LA. Correlation Coefficients: Appropriate Use and Interpretation. *Anesth Analg* 2018; 126: 1763-1768.

7. Global Initiative for Chronic Obstructive Lung Disease (GOLD). 2023. Available from: https://goldcopd.org/2023-gold-report-2/.

8. Douglas JG, Middleton WG, Gaddie J, Petrie GR, Choo-Kang YF, Prescott RJ, Crompton GK. Sarcoidosis: a disorder commoner in non-smokers? *Thorax* 1986; 41: 787-791.

9. Handa T, Nagai S, Fushimi Y, Miki S, Ohta K, Niimi A, Mishima M, Izumi T. Clinical and radiographic indices associated with airflow limitation in patients with sarcoidosis. *Chest* 2006; 130: 1851-1856.

10. Terasaki H, Fujimoto K, Muller NL, Sadohara J, Uchida M, Koga T, Aizawa H, Hayabuchi N. Pulmonary sarcoidosis: comparison of findings of inspiratory and expiratory high-resolution CT and pulmonary function tests between smokers and nonsmokers. *AJR Am J Roentgenol* 2005; 185: 333-338.

11. Gupta D, Singh AD, Agarwal R, Aggarwal AN, Joshi K, Jindal SK. Is tobacco smoking protective for sarcoidosis? A case-control study from North India. *Sarcoidosis Vasc Diffuse Lung Dis* 2010; 27: 19-26.
